# Clinical Relevance of the HALP Score in Rheumatoid Arthritis: Association with Disease Activity and Inflammatory Burden

**DOI:** 10.5152/ArchRheumatol.2026.25181

**Published:** 2026-03-13

**Authors:** Gülşah Çelik, Çiğdem Çilingiroğlu, Sevcan Uğur, Şevval Dirican Kızıl

**Affiliations:** 1 Department of Physical Medicine and Rehabilitation, Ceylanpınar State Hospital, Şanlıurfa, Türkiye; 2 Department of Physical Medicine and Rehabilitation, Antalya City Hospital, Antalya, Türkiye; 3 Department of Rheumatology, Antalya City Hospital, Antalya, Türkiye

**Keywords:** Biomarker, disease activity, HALP score, inflammation, rheumatoid arthritis

## Abstract

**Background/Aims:**

The hemoglobin, albumin, lymphocyte, and platelet (HALP) score is a novel biomarker reflecting systemic inflammation and nutritional status. While it has been studied in oncology and some inflammatory diseases, its clinical role in rheumatoid arthritis (RA) remains unclear. This study aimed to investigate the relationship between HALP, disease activity, and inflammatory burden in RA.

**Materials and Methods:**

This retrospective cross-sectional study included 106 patients with RA diagnosed according to the 2010 ACR/EULAR criteria and 110 age-, sex-, and BMI-matched healthy controls. Clinical and laboratory data were recorded, and HALP scores were calculated. Disease activity was assessed using the disease activity score-28-C-reactive protein (DAS28-CRP). Group comparisons, Spearman correlation analysis, receiver-operating characteristic (ROC) curve analysis, and multivariable logistic regression were performed.

**Results:**

The HALP scores were significantly lower in patients with RA compared with healthy controls (*P* < .0001). Among patients with RA, those with active disease exhibited significantly lower HALP scores than those in remission (*P* < .05), with a decreasing trend across DAS28-CRP disease activity categories. The HALP scores showed negative correlations with CRP (*r* = −0.329), Visual Analog Scale (VAS)-pain (*r *= −0.399), and DAS28-CRP (*r* = −0.348) (all *P* ≤ .001). The ROC analysis demonstrated moderate discriminative ability for distinguishing active disease from remission (AUC = 0.708, 95% CI: 0.63-0.81). In multivariable logistic regression analysis, lower HALP scores and older age were independently associated with active RA.

**Conclusion:**

The study demonstrates that lower HALP scores are associated with higher disease activity in rheumatoid arthritis. As a simple and routinely available laboratory-based index, the HALP score may serve as a supportive marker of inflammatory burden. Prospective multicenter studies are required to validate these findings and to clarify the longitudinal utility of the HALP score.

**Main Points**

- The hemoglobin, albumin, lymphocyte, and platelet (HALP) scores were significantly lower in patients with active rheumatoid arthritis (RA) compared with those in remission.
- The HALP scores showed an inverse association with established measures of disease activity, including C-reactive protein (CRP), Visual Analog Scale (VAS) pain, and C-reactive protein-CRP (DAS28-CRP).
- The HALP scores show a decreasing trend across DAS28-CRP disease activity categories.
- The HALP scores demonstrate moderate discriminatory ability between active disease and remission.
- The HALP scores may serve as a supportive laboratory-based marker of inflammatory burden in RA.
- This study provides novel evidence linking HALP with disease activity in RA.

## Introduction

Rheumatoid arthritis (RA) is characterized as a persistent autoimmune condition with systemic involvement that predominantly presents as symmetrical, bilateral polyarthritis.^1^ Although its prevalence and incidence differ among populations, RA is estimated to affect approximately 0.5%-1% of individuals worldwide, with recent meta-analyses reporting a global prevalence of around 0.46%.^2^^,^^3^ In advanced or poorly controlled cases, it may lead to irreversible joint damage, substantial functional limitations, and impaired psychological and social well-being, thereby contributing to increased morbidity and mortality.^4^

Rheumatoid arthritis is mainly identified through internationally recognized diagnostic frameworks, among which the 2010 American College of Rheumatology/European League Against Rheumatism (ACR/EULAR) criteria are the most commonly applied in clinical settings.^5^ These criteria combine clinical findings, serological markers, and duration of symptoms to identify patients with RA at early stages and guide treatment decisions.

To evaluate disease activity and monitor therapeutic response, several composite indices have been developed, including the disease activity score based on 28 joints (DAS28), the Clinical Disease Activity Index (CDAI), and the Simplified Disease Activity Index (SDAI).^6^ Although these tools are highly valid, they include subjective measures like the number of tender joints and patients’ overall self-assessments, which may be influenced by observer differences and individual perception bias.

In addition to clinical scoring systems, inflammatory biomarkers such as erythrocyte sedimentation rate (ESR) and C-reactive protein (CRP) are routinely used to quantify disease activity and support clinical judgment.^7^ However, despite their widespread use, these markers are limited by their low specificity and short-term reflection of inflammatory burden, particularly in the presence of other inflammatory or infectious conditions.^8^

The overarching goals in RA management are to suppress inflammation, prevent joint destruction, and preserve functional capacity. Achieving these goals requires regular, objective, and reliable monitoring of disease activity from the earliest stages, as irreversible joint damage can occur rapidly.^9^ The partial subjectivity of clinical indices and the limitations of current laboratory markers underscore the need for an accessible, reproducible, and comprehensive biomarker that can support disease monitoring and provide additional information on systemic inflammation in RA.^10^

The hemoglobin, albumin, lymphocyte, and platelet (HALP) score is a composite index that integrates hematological and biochemical parameters to provide a combined assessment of a patient’s inflammatory and nutritional status.^11^^-^^13^ It is calculated using the formula: serum albumin (g/L) × hemoglobin (g/L) × lymphocyte count (/L) ÷ platelet count (/L).^14^ The individual parameters within the HALP score offer specific clinical information: hemoglobin reflects anemia status, which is frequently exacerbated by chronic inflammation; albumin, a negative acute-phase reactant, decreases in systemic inflammatory states and has traditionally been regarded as a marker of nutritional status; lymphocyte count provides insight into immune competence, with lower levels suggesting impaired immunity; and platelet count often increases in the presence of inflammation, reflecting heightened thrombotic and immune activity.^11^^,^^15^

Inflammation-related laboratory markers such as CRP, leukocytes, platelets, ferritin, and albumin are known to fluctuate in systemic inflammatory diseases including malignancies and autoimmune disorders like ankylosing spondylitis (AS).^16^ In recent years, the HALP score has been examined regarding its potential clinical relevance and prognostic implications across diverse clinical contexts. Multiple studies have demonstrated its association with survival outcomes in colorectal, bladder, gastric, and renal cancers, as well as its potential prognostic utility in chronic inflammatory conditions.^16^^-^^20^ Moreover, research in anti-neutrophil cytoplasmic antibody–associated vasculitis has suggested that the HALP score could serve as a supportive index associated with disease activity.^18^

When these factors are brought together, the HALP score may serve as an easy-to-use and comprehensive indicator of systemic inflammation. Recent studies have demonstrated the prognostic utility of the HALP score in various inflammatory and malignant conditions. Current evidence suggests that the clinical significance of the HALP score in patients with RA remains unexplored. This study is, therefore, the first to investigate the clinical relevance of the HALP score in RA and to examine its association with systemic inflammation and disease activity.

## Materials and Methods

### Study Design and Participitans

In this retrospective, cross-sectional design, 106 patients were enrolled, all of whom had been monitored at the Physical Medicine and Rehabilitation and Rheumatology outpatient clinics of Antalya City Hospital between January 2024 and December 2024, with RA diagnosed according to the 2010 ACR classification criteria.^5^ The control group comprised 110 healthy individuals who were matched to the patient group with respect to demographic characteristics, had no evidence of systemic, inflammatory, or malignant disease, and showed normal routine laboratory findings. These individuals were selected from among those presenting to the hospital for routine health check-ups.

Patients were excluded if they had missing clinical or laboratory data; a diagnosis of other connective tissue diseases such as systemic lupus erythematosus, Sjögren’s syndrome, or systemic sclerosis; a history of malignancy or active cancer; acute or chronic infections (e.g., tuberculosis, viral infections, sepsis); hepatic or renal diseases such as cirrhosis or chronic renal failure; current or former smoking; pregnancy or breastfeeding; or chronic systemic comorbidities not attributable to RA that could independently affect inflammatory parameters (e.g., diabetes mellitus, non-RA cardiovascular diseases, or primary neurological disorders such as prior stroke or multiple sclerosis).

With respect to treatment characteristics of the study population, in this retrospective study, biologic therapy was prescribed in accordance with current clinical practice guidelines, as documented in medical records. Patients receiving biologic agents had persistent disease activity despite conventional DMARD therapy, as assessed by clinical findings and inflammatory markers.

### Ethical Approval

Approval for the conduct of this research was granted by the Clinical Research Ethics Committee of Antalya City Hospital (Approval No: 1/8, Date: January 30, 2025). This research was carried out following the ethical principles outlined in the 1964 Declaration of Helsinki. As the study was retrospective in nature and based on medical records, informed consent from individual participants was not required. Patient data were anonymized and confidentiality was strictly maintained.

### Outcome Assessments

Demographic and clinical characteristics, including age, sex, BMI, disease duration, presence of extra-articular involvement, serological test results (rheumatoid factor (RF), anti-cyclic citrullinated peptide (anti-CCP) antibodies), and medications used, were recorded.

Laboratory parameters included hemoglobin (g/L), albumin (g/L), lymphocyte count (/L), and platelet count (/L), ESR (mm/h; measured by the Westergren method), and CRP (mg/L; measured by turbidimetric method). Rheumatoid factor was measured using nephelometry, with values > 15 IU/mL considered positive. The detection of anti-CCP antibodies was performed through an enzyme-linked immunosorbent assay method, considering concentrations above 20 U/mL as indicative of positivity.

Blood samples were obtained during routine clinical evaluation. Complete blood count parameters were measured from potassium-ethylenediaminetetraacetic acid tubes without centrifugation using a fluorescence flow cytometry–based automated analyzer (XN-1000, Sysmex Corporation, Kobe, Japan). For biochemical analyses, blood samples were collected into gel separator tubes and centrifuged at 1800 × g for 10 minutes (or 4000 rpm for 10 minutes). Serum CRP levels were measured using a turbidimetric method, and serum albumin levels were determined using a photometric method on a closed-system analyzer (Cobas 8000 series c702, Roche Diagnostics, Mannheim, Germany).

Pain intensity was assessed using a 100-mm Visual Analog Scale (VAS), where 0 represented no pain and 100 indicated the worst imaginable pain.^21^

Disease activity was evaluated with the Disease Activity Score-28 based on CRP (DAS28-CRP), incorporating the tender joint count (TJC28), swollen joint count (SJC28), patient global health assessment measured on a 0-100 mm VAS-pain, and CRP level. The DAS28-CRP score was calculated using the validated formula. Patients were categorized into 4 disease activity states according to established cut-off values: remission (DAS28-CRP ≤ 2.6), low disease activity (DAS28-CRP > 2.6 to ≤ 3.2), moderate disease activity (DAS28-CRP > 3.2 to ≤ 5.1), and high disease activity (DAS28-CRP > 5.1).^6^^,22^ Joint counts were assessed by a rheumatologist with expertise, who was unaware of the laboratory results.

The HALP score was calculated as follows: hemoglobin (g/L) × albumin (g/L) × lymphocyte count (/L) ÷ platelet count (/L) in accordance with previously published methodology.^14^

Extra-articular involvement was defined as the presence of organ or tissue manifestations beyond the joints (e.g., ocular, pulmonary, cutaneous, cardiovascular) confirmed by clinical evaluation, imaging, or histopathological findings.

### Statistical Analysis

Descriptive statistics were presented as frequencies, percentages, means, standard deviations, medians, and 25th (Q1) and 75th (Q3) percentiles. For categorical variables, Fisher’s exact test was used when more than 20% of the cells had expected counts less than 5; otherwise, the Pearson chi-square test was applied. When statistically significant differences were detected, column proportions were compared using the *z*-test, and Bonferroni correction was applied for multiple comparisons. The normality of continuous variables was assessed using the Shapiro–Wilk test. As the numerical data did not follow a normal distribution, differences between 2 groups were analyzed using the Mann–Whitney *U-*test. Associations between numerical variables were evaluated using the Spearman rank correlation coefficient. All statistical analyses were performed using SPSS version 23.0 (IBM SPSS Corp.; Armonk, NY, USA), and a *P*-value < .05 was considered statistically significant. To identify independent factors associated with active RA, a multivariable logistic regression analysis was performed, and variables considered clinically relevant were included in the model. Results were reported as odds ratios (ORs) with 95% confidence intervals. Model fit was assessed using the Hosmer–Lemeshow test, and model explanatory power was evaluated using the Cox & Snell and Nagelkerke R² coefficients. Classification performance was assessed based on the overall correct classification rate (%). To evaluate the discriminative performance of the HALP score, receiver-operating characteristic (ROC) curve analysis was conducted. The Youden index was used to determine the optimal cut-off value. The area under the ROC curve (AUC) was calculated to assess discriminatory ability, and results were reported with 95% confidence intervals. The ROC curve analyses were performed using MedCalc Statistical Software v22.009 (trial version) (MedCalc Software Ltd; Ostend, Belgium) .^23^

## Results

A total of 216 participants were included in the study (106 patients with RA and 110 healthy controls). There were no significant differences between the groups with respect to age, sex, or BMI (all *P* > .05). In the RA group, the mean disease duration was 66.85 ± 52.78 months, and 66% of patients had active disease according to DAS28-CRP criteria. The Anti-CCP and RF positivity were observed in 62.3% and 66% of patients, respectively. Compared with healthy controls, patients with RA had significantly lower hemoglobin, lymphocyte, albumin, and HALP scores, while platelet counts, CRP, and ESR levels were significantly higher (all *P* < .05; Table 1).

When patients with RA were stratified according to disease activity, HALP scores were significantly lower in patients with active disease than in those in remission (*P* < .05). Moreover, HALP scores showed a decreasing trend across DAS28-CRP disease activity categories. Patients with higher disease activity were older and had lower hemoglobin levels and higher platelet counts and inflammatory markers (CRP and ESR), whereas BMI, sex distribution, disease duration, extra-articular involvement, medication use, and anti-CCP positivity did not differ significantly between activity groups (Table 2).

Spearman correlation analysis showed that HALP scores were negatively correlated with CRP (*r *= −0.329, *P* = .001), VAS-pain (*r *= −0.399, *P* < .0001), and DAS28-CRP (*r* = −0.348, *P* < .0001), while no significant correlation was observed with ESR or disease duration (Table 3).

**Figure 1.**
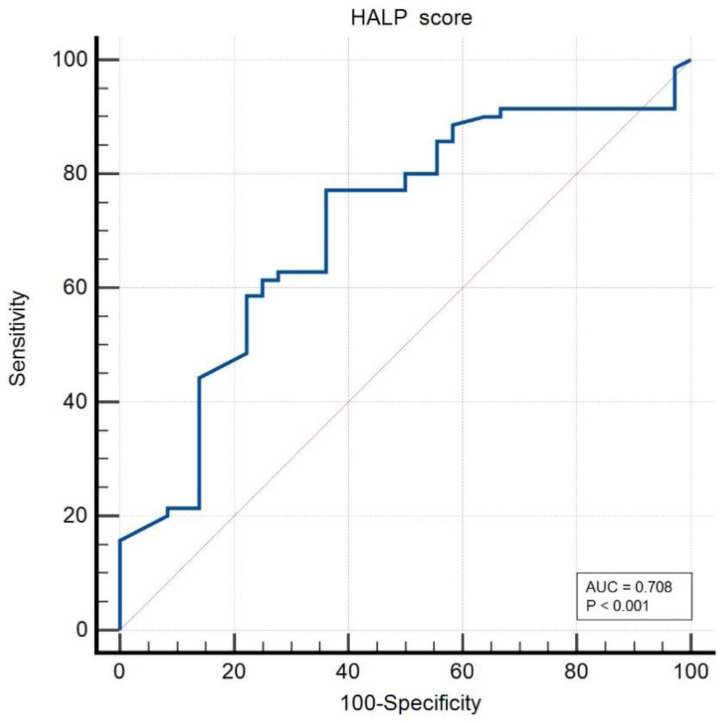
ROC curve of HALP Score.

The ROC analysis demonstrated that HALP could discriminate between active disease and remission with moderate accuracy (AUC = 0.708, 95% CI: 0.63-0.81; *P* < .001). Using a cut-off value of ≤41.6, sensitivity and specificity were 77.1% and 63.9%, respectively (Table 4, Figure 1).

In multivariable logistic regression analysis, both age and HALP score were independently associated with active RA. Increasing age was associated with higher odds of active disease (OR = 1.041, 95% CI: 1.002-1.082; *P* = .041), while lower HALP scores were independently associated with active disease (OR = 0.949, 95% CI: 0.919-0.981; *P* = .002) (Table 5).

## Discussion

In this study, the HALP score was consistently lower in patients with RA compared to healthy controls and was inversely associated with clinical and laboratory indicators of disease activity. Lower HALP values were particularly evident in patients with active disease. The ROC analysis further suggested that HALP can distinguish active disease from remission with moderate accuracy, supporting its interpretation as a marker of inflammatory burden rather than a diagnostic test. Overall, HALP may provide complementary, objective information when interpreted alongside established indices such as DAS28-CRP.

In multivariable logistic regression, lower HALP scores remained independently associated with active RA after adjustment for relevant covariates. This finding suggests that HALP captures aspects of systemic inflammation that are not fully reflected by demographic or clinical variables alone. Age also emerged as an independent factor associated with active disease, which is consistent with the cumulative inflammatory burden observed in older RA populations and further supports the robustness of the association between HALP and disease activity. Given that active RA is commonly accompanied by anemia of chronic inflammation, hypoalbuminemia, immune cell alterations, and reactive thrombocytosis, the composite nature of HALP may offer an integrated snapshot of the systemic inflammatory milieu. In addition, HALP scores demonstrated a graded decline across increasing DAS28-CRP disease activity categories, indicating that HALP is sensitive to varying levels of disease activity rather than merely distinguishing between remission and active disease.

The HALP score is a composite index that integrates routine hematological and biochemical parameters into a single, cost-effective measure of inflammation.^13^^,^^19^ Originally developed in oncology, it has been validated as a prognostic indicator in various malignancies and increasingly investigated in non-malignant inflammatory conditions.^24^^-^^26^ Each component reflects distinct pathophysiological changes in RA. Reduced hemoglobin may exacerbate hypoxia and tissue damage, while albumin, a negative acute-phase reactant, decreases in response to inflammatory cytokines.^27^^,^^28^ Lymphocyte depletion may occur through cytokine-driven dysregulation of apoptosis.^29^ Platelets can increase in response to systemic inflammation, driven by elevated inflammatory cytokines, and contribute to the inflammatory milieu.^30^ Given that RA is characterized by persistent inflammation, these hematological and biochemical alterations captured by the HALP formula provide an integrated view of the patient’s systemic inflammatory status.^31^ In the study, while all 4 components of HALP differed significantly between RA and control groups, only lymphocyte count did not show a statistically significant difference between active and remission subgroups. The relatively small number of cases in the subgroup could have lessened the statistical capacity to reveal subtle distinctions, despite reduced median lymphocyte levels in the active group.

Traditional inflammatory markers such as ESR and CRP are well-established tools for evaluating disease activity in RA; however, they are influenced by non-inflammatory factors and temporal variability and may not always accurately reflect clinical disease activity.^32^^-^^34^ Although VAS-pain is a subjective measure, pain perception in RA is influenced by the overall inflammatory milieu.^38^ Systemic inflammation can contribute to increased pain perception through inflammatory cytokine activity, anemia of chronic disease, and alterations in nutritional and hematological parameters, which may also be captured by the HALP score. Patients with active disease may therefore report higher pain levels even when objective joint swelling is limited. Accordingly, the observed negative correlation between HALP and VAS-pain may reflect an association between systemic inflammatory burden and patient-reported symptom severity. In contrast to single inflammatory markers, HALP integrates multiple routine hematologic and biochemical parameters and may provide complementary, objective information alongside DAS28, which relies partly on joint counts and patient-reported outcomes.

The findings are consistent with prior work suggesting lower HALP values in inflammatory conditions. Antar et al reported lower HALP values among individuals with arthritis compared to those without arthritis in a population-based dataset; however, their reliance on self-reported diagnoses and heterogeneous arthritis phenotypes may have attenuated disease-specific associations.^11^ By contrast, this study included clinically confirmed RA cases using the 2010 ACR/EULAR criteria and demonstrated a clear relationship between HALP and RA disease activity. In addition, studies in other chronic inflammatory rheumatic diseases (e.g., AS) and systemic vasculitides have similarly shown lower HALP levels in active disease, supporting the concept that HALP reflects systemic inflammatory activity across immune-mediated disorders, although disease-specific characteristics may influence optimal cut-off values and performance metrics.^16^^,^^18^

The prognostic value of HALP has been widely investigated in non-rheumatologic conditions, particularly malignancies.^13^^,^^35^^39-40^ Previous studies have shown that low HALP levels are associated with adverse outcomes across several cancer types, and in some settings, HALP has been reported to outperform traditional tumor markers.^15^^,^^36^ These findings support the broader applicability of HALP as a composite marker integrating erythropoiesis, albumin metabolism, immune status, and platelet-driven inflammation, processes that are also relevant to chronic inflammatory diseases such as RA.

Hematological indices such as the neutrophil-to-lymphocyte ratio and platelet-to-lymphocyte ratio have been proposed as inexpensive inflammatory markers in RA; however, they primarily reflect leukocyte and platelet dynamics and may be influenced by transient physiological changes or treatment effects.^8^^,^^10^^,^^37^^41^ In contrast, the HALP score integrates hemoglobin, albumin, lymphocyte count, and platelet count, thereby providing a more comprehensive laboratory-based representation of systemic inflammation. This multidimensional nature may account for its association with disease activity observed in the present study.

From a clinical perspective, HALP could be a valuable adjunct in monitoring RA patients, particularly in settings where comprehensive disease activity scoring is not feasible. Its reliance on widely available laboratory parameters enables use even in resource-limited environments, and its integration into routine blood panels could facilitate regular, cost-effective assessment of systemic inflammation.

Strengths of the study include the use of clinically confirmed RA diagnoses based on the 2010 ACR/EULAR criteria, exclusion of smokers to reduce hematological confounding, and simultaneous evaluation of HALP components and established inflammatory markers.

The study has several limitations. Its retrospective, single-center design limits the generalizability of the findings, and the cross-sectional nature of the study precludes assessment of longitudinal changes in HALP scores or their response to treatment. Although major confounding factors such as smoking and overt systemic diseases were excluded, hemoglobin and albümin, key components of the HALP score, may still be influenced by factors unrelated to RA activity, including nutritional status, subclinical infections, and concomitant medications (e.g., corticosteroids or immunosuppressants), which were not systematically evaluated. In addition, dietary intake and validated nutritional assessment data were unavailable, which may have affected HALP values independently of inflammatory burden.

Another limitation is the lack of direct long-term comparison between HALP and conventional inflammatory markers such as CRP and ESR, which would be valuable in further defining its clinical utility. Furthermore, the exclusion of patients with a smoking history, active infections, malignancy, other rheumatic diseases, and chronic systemic comorbidities, while methodologically necessary to reduce confounding, may limit the applicability of the findings to real-world RA populations, where such factors are common. Information regarding alcohol consumption was also unavailable due to the retrospective design.

Finally, treatment data reflected medications used at the time of evaluation and may not fully capture prior treatment exposure or escalation. Disease activity was assessed using DAS28-CRP, which may classify patients as having active disease despite low swollen joint counts when pain-related components or systemic inflammation predominate. Consequently, the study population may predominantly represent patients with mild to moderate RA, limiting generalizability to severe disease.

The study demonstrates that the HALP score is lower in patients with active RA and is inversely associated with disease activity. The HALP score may serve as a supportive marker of inflammatory burden. Prospective, multicenter studies are required to validate these findings and to clarify the longitudinal utility of the HALP score.

## Figures and Tables

**Table 1.** Bold values indicate statistically significant differences (*P* < .05).

	**Control Group (n = 110)**	**RA Group (n = 106)**	*P*
Age (years), median (Q1-Q3)	54.5(46-62)	58(50-64)	.157^1^
BMI (kg/m^2^), median (Q1-Q3)	25.71(24.84-27.55)	25.94(23.78-28.08)	.697^1^
Sex, n (%)			
Female	82(74.50)	80(75.50)	.875^2^
Male	28(25.50)	26(24.50)	
Disease duration (months), mean ± SD-		66.85 ± 52.78	
Extra-articular involvement, n (%)			
Present Peripheral neuropathy, n (%) Interstitial lung disease, n (%) Episcleritis / scleritis, n (%) Vasculitis, n (%)		9(8.5) 4(3.8) 3(2.9) 1(0.9) 1(0.9)	
Absent		97(91.5)	
Medication use, n ((%)			
NSAIDs		23(21.7)	
DMARDs Methotrexate, n (%) Leflunomide, n (%) Hydroxychloroquine, n (%) Sulfasalazine, n (%)		63(59.4) 28(26.4) 23(21.7) 8(7.5) 4(3.8)	
Biologic agents TNF inhibitors, n (%) Non-TNF biologic agents, n (%)		20(18.9) 15(14.1) 5(4.8)	
anti-CCP, n (%)			
Positive		66(62.3)	
Negative		40(37.7)	
RF, n (%)			
Positive		70(66)	
Negative		36(34)	
Disease activity, n (%)			
Remission		36(34)	
Active		70(66)	
Laboratory parameters			
Hemoglobin (g/dL), median (Q1-Q3)	13.6(13-14.5)	12.7(11.4-13.2)	***<.0001^1^***
Lymphocytes (×10³/μL), median (Q1-Q3)	2.33(1.91-2.7)	2.10(1.6-2.5)	***.005^1^***
Platelets (×10³/μL), median (Q1-Q3)	276(243-305)	299(241-343)	***.015^1^***
Albumin (g/dL), median (Q1-Q3)	4.5(4.4-4.7)	4.1(3.9-4.4)	***<.0001^1^***
HALP score, median (Q1-Q3)	51.85(42.22-62.68)	36.94(26.36-46.63)	***<.0001^1^***
CRP (mg/L), median (Q1-Q3)	2.5(1-3.9)	8(5.3-16.2)	***<.0001^1^***
ESR (mm/h), median (Q1-Q3)	9(4-15)	18(12-33)	***<.0001^1^***
Tender joint, mean ± SD		3.11 ± 4.08	
Swollen joint, mean ± SD		0.97 ± 2.11	
VAS-pain, mean ± SD		52.65 ± 25.72	
DAS-28, mean ± SD		3.37 ± 1.16	

BMI, body mass index; CCP, cyclic citrullinated peptide; CRP, C-reactive protein; DAS, disease activity score; DMARDs, disease-modifying antirheumatic drugs; ESR, erythrocyte sedimentation rate; HALP, hemoglobin, albumin, lymphocyte, platelet; NSAIDs, non-steroidal anti-inflammatory drugs; RF, rheumatoid factor; SD, standard deviation; VAS, Visual Analog Scale; Q1, first quartile (25th percentile); Q3, third quartile (75th percentile).

¹Mann–Whitney *U-*test; ²Pearson’s chi-square test was used.

Bold values indicate statistically significant differences (*P* < .05).

**Table 2.** Comparison of Clinical and Laboratory Characteristics According to Disease Activity Categories in Patients with Rheumatoid Arthritis

	**R (n = 36)**	**LDA (n = 16)**	**MDA (n = 45)**	**HDA (n = 9)**	*P*
Age (years), median (Q1-Q3)	56(50-63)^b^	59.5(45.5-66)^ab^	59(50-68)^a^	63(54-68)^b^	***.008*** ***^2^***
BMI (kg/m^2^), median (Q1-Q3)	26.3(23.23-28.39)	27.39(20.31-35.16)	25.95(19.47-35.94)	26.47(24.72-28.65)	.329^1^
Sex, n (%)					
Female	28(77.8)	9(56.3)	36(80)	7(77.8)	.321^4^
Male	8(22.2)	7(43.8)	9(20)	2(22.2)	
Disease duration (months), mean ± SD	72(15-108)	75(42-109)	48(12-108)	72(48-100)	.413^2^
Extra-articular involvement, n(%)					
Present	3(8.3)	3(18.8)	3(6.6)	0(0)	.460^4^
Absent	33(91.7)	13(81.2)	42(93.4)	9(100)	
Medication use, n (%)					
NSAIDs	6(16.7)	2(12.5)	12(26.6)	3(33.3)	.722^4^
DMARDs	22(61.1)	12(75)	25(55.5)	4(44.5)	
Biologic agents	8(22.2)	2(12.5)	8(17.9)	2(22.2)	
anti-CCP, n (%)					
Positive	24(66.7)	12(75)	27(60)	3(33.3)	.221^3^
Negative	12(33.3)	4(25)	18(40)	6(66.7)	
RF, n (%)					
Positive	24(66.7)^ab^	15(93.8)^b^	28(62.2)^ab^	3(33.3)^a^	***.019*** ***^3^***
Negative	12(33.3)^ab^	1(6.3)^b^	17(37.8)^ab^	6(66.7)^a^	
Laboratory parameters					
Hemoglobin (g/dL), median (Q1-Q3)	13.2(12.35-13.45)^a^	12.4(11.55-13.2)^ab^	12.4(11.4-13.2)^ab^	11.4(11.1-12.7)^b^	***.027*** ***^2^***
Lymphocytes (×10³/μL), median (Q1-Q3)	2.15(1.6-2.4)	2.05(1.5-2.55)	2.1(1.6-2.4)	2.4(1.9-2.5)	.479^2^
Platelets (×10³/μL), median (Q1-Q3)	270.5(234-307.5)^b^	296(258.5-329.5)^ab^	301(249-350)^ab^	367(308-383)^a^	***.005*** ***^2^***
Albumin (g/dL), median (Q1-Q3)	4.2(4.05-4.4)	4.2(3.9-4.4)	4(3.7-4.3)	4(3.8-4.3)	.100^2^
HALP score, median (Q1-Q3)	44.03(35.99-56.17)^a^	31.79(24.85-47.72)^b^	30.35(25.05-41.4)^b^	29.83(21.08-40.86)^b^	***.001*** ***^2^***
CRP (mg/L), median (Q1-Q3)	5.35(1.6-7.7)^c^	5.95(2.1-10.85)^c^	11.6(8-24.2)^b^	49.7(24.2-66.4)^a^	***<.001*** ***^2^***
ESR (mm/h), median (Q1-Q3)	14.5(6-30.5)^ab^	12.5(6-26)^b^	20(13-39)^ab^	29(18-44)^a^	***.047*** ***^2^***
Tender joint, mean ± SD	0(0-0)^c^	1(0-2)^c^	3(1-8)^b^	10(8-11)^a^	***<.001*** ***^2^***
Swollen joint, mean ± SD	0(0-0)^b^	0(0-0)^b^	0(0-2)^b^	1(0-3)^a^	***.004*** ***^2^***
VAS-pain, mean ± SD	30(13.5-50)^c^	50(40-55)^b^	50(50-80)^b^	90(90-90)^a^	***<.001*** ***^2^***
DAS-28, mean ± SD	2.12(1.92-2.44)^c^	2.99(2.7-3.08)^c^	4.06(3.45-4.48)^b^	5.66(5.49-5.73)^a^	***<.001*** ***^2^***

BMI, body mass index; CCP, cyclic citrullinated peptide; CRP, C-reactive protein; DAS, disease activity score; DMARDs, disease-modifying antirheumatic drugs; ESR, erythrocyte sedimentation rate; HALP, hemoglobin, albumin, lymphocyte, platelet; HDA, High Disease Activity; LDA, Low Disease Activity; MDA, Moderate Disease Activity; NSAIDs, non-steroidal anti-inflammatory drugs; R, remisson; RF, rheumatoid factor; SD, standard deviation; VAS, Visual Analog Scale; Q1, first quartile (25th percentile); Q3, third quartile (75th percentile).

Groups with different superscript letters differ significantly (*P* < .05).

^1^One way ANOVA, ^2^Kruskal Wallis H, ^3^Pearson chi squred, ^4^Fisher exact test were used. Pairwise comparisons were performed using Dunn-Bonferroni post hoc procedure.

Bold values indicate statistically significant differences (*P* < .05).

**Table 3.** Relationship between HALP Score and Disease Duration, CRP, ESR, VAS, and DAS-28 in Patients with Rheumatoid Arthritis

	**HALP Score**	
	*r*	*P*
Disease duration (months)	−0.117	.232
CRP (mg/L)	−.329**	**.001**
ESR (mm/h)	−0.171	0.08
VAS-pain	−.399**	**<.0001**
DAS-28	−.348**	**<.0001**

CRP, C-reactive protein; DAS, disease activity score; ESR, erythrocyte sedimentation rate; HALP, hemoglobin, albumin, lymphocyte, platelet; VAS, Visual Analog Scale; *r*, Spearman’s rank corelation coefficient.

**Statistically significant at .01.

Bold values indicate statistically significant differences (*P* < .05).

**Table 4.** ROC Analysis of HALP Score for Discriminating Active Rheumatoid Arthritis from Remission

	**AUC (%95 CI)**	*P*	**Cut-off**	**Sensitivity**	**Specificity**	**Youden Index**
HALP Score	0.708 (0.63-0.813)	<.001	≤41.6	77.14	63.89	0.410

HALP, hemoglobin, albumin, lymphocyte, platelet.

**Table 5.** Multivariable Logistic Regression Analysis of Factors Associated with Active Rheumatoid Arthritis

**Variables**	**B**	**SE**	**Wald**	*P*	** Exp(B)**	**%95 CI**
Age (years)	0.040	0.020	4.185	***.041***	1.041	1.002-1.082
Sex (female vs male)	0.490	0.561	0.762	.383	1.632	0.543-4.901
BMI (kg/m²)	0.029	0.063	0.211	.646	1.029	0.910-1.164
Disease duration (months)	-0.006	0.004	2.137	.144	0.994	0.986-1.002
HALP score	-0.052	0.017	9.821	***.002***	0.949	0.919-0.981

Hosmer–Lemeshow test: χ²=7.603; *P* = .473; -2LL=119.064; Cox & Snell R²=0.146; Nagelkerke R²=0.203; Classification rate: 69.8 %.

CI, confidence interval; OR, odds ratio; SE, standard error.

Bold values indicate statistically significant differences (*P* < .05).

## Data Availability

The data that support the findings of this study are available on request from the corresponding author.
